# Identification of Novel HLA Class II-Restricted Neoantigens Derived from Driver Mutations

**DOI:** 10.3390/cancers11020266

**Published:** 2019-02-24

**Authors:** Susumu Iiizumi, Junya Ohtake, Naoko Murakami, Taku Kouro, Mamoru Kawahara, Fumiko Isoda, Hiroshi Hamana, Hiroyuki Kishi, Norihiro Nakamura, Tetsuro Sasada

**Affiliations:** 1 Department of Cancer Immunotherapy, Kanagawa Cancer Center Research Institute, 2-3-2 Nakao, Asahi-ku, Yokohama, Kanagawa 241-8515, Japan; iizumi_s@brightpathbio.com (S.I.); qsl.suivx2000@gmail.com (J.O.); n.murakami_0131v2@outlook.jp (N.M.); kourot@gancen.asahi.yokohama.jp (T.K.); mmrkawahara@gancen.asahi.yokohama.jp (M.K.); isoda_f@brightpathbio.com (F.I.); 2 Research & Early Development Division, BrightPath Biotherapeutics Co., Ltd., 3-25-22 Tonomachi, Kawasaki-ku, Kawasaki, Kanagawa 210-0821, Japan; nakamura_n@brightpathbio.com; 3 Department of Innovative Cancer Immunotherapy, Graduate School of Medicine and Pharmaceutical Sciences, University of Toyama, 2630 Sugitani, Toyama 930-0194, Japan; hamana@med.u-toyama.ac.jp; 4 Department of Immunology, Graduate School of Medicine and Pharmaceutical Sciences, University of Toyama, 2630 Sugitani, Toyama 930-0194, Japan; immkishi@med.u-toyama.ac.jp

**Keywords:** neoantigen, driver mutation, MHC class II epitope

## Abstract

Neoantigens derived from tumor-specific genetic mutations might be suitable targets for cancer immunotherapy because of their high immunogenicity. In the current study, we evaluated the immunogenicity of 10 driver mutations that are frequently expressed in various cancers using peripheral blood mononuclear cells from healthy donors (*n* = 25). Of the 10 synthetic peptides (27-mer) derived from these mutations, the six peptides from KRAS-G12D, KRAS-G12R, KRAS-G13D, NRAS-Q61R, PIK3CA-H1047R, and C-Kit-D816V induced T cell responses, suggesting that frequent driver mutations are not always less immunogenic. In particular, immune responses to PIK3CA-H1047R, C-Kit-D816V, KRAS-G13D, and NRAS-Q61R were observed in more than 10% of the donors. All six peptides induced human leukocyte antigen (HLA) class II-restricted CD4^+^ T cell responses; notably, PIK3CA-H1047R contained at least two different CD4^+^ T cell epitopes restricted to different HLA class II alleles. In addition, PIK3CA-H1047R and C-Kit-D816V induced antigen-specific CD8^+^ T cells as well, indicating that they might contain both HLA class I- and class II-restricted epitopes. Since the identified neoantigens might be shared by patients with various types of cancers and are not easily lost due to immune escape, they have the potential to be promising off-the-shelf cancer immunotherapy targets in patients with the corresponding mutations.

## 1. Introduction

Numerous clinical trials of cancer immunotherapies, such as cancer vaccines, have been performed for the induction and amplification of immune responses to tumor-associated antigens (TAAs) [1,2]. Unfortunately; however, most of these trials have failed to demonstrate therapeutic benefits [2,3]. Such results may be explained, at least in part, by the TAAs employed, because most of such TAAs are derived from non-mutated self-antigens [2,3,4], which cannot be expected to show high immunogenicity because of the central and/or peripheral tolerance mechanism.

Recently, increasing attention has been paid to neoantigens that are derived from somatic genetic mutations specifically present in cancer cells. Since they can be recognized as non-self by the immune system, they are expected to induce stronger immune responses than non-mutated self-antigens [5,6]. In particular, since “driver mutations” that are directly involved in malignant processes are frequently shared by patients with various types of cancers and do not disappear easily by immune escape, they could represent appropriate off-the-shelf cancer immunotherapy targets. However, limited information is available regarding the immunogenicity of frequent driver mutations.

In the current study, we examined human leukocyte antigen (HLA) class I- and class II-restricted T cell responses to 10 driver mutations that are present in various cancer types using a panel of long synthetic peptides and peripheral blood mononuclear cells (PBMCs) from normal healthy donors. Our findings suggested that immunogenic driver mutations, including KRAS-G12D, KRAS-G12R, KRAS-G13D, NRAS-Q61R, PIK3CA-H1047R, and C-Kit-D816V, have the potential to be promising off-the-shelf tumor immunotherapy targets in patients with the corresponding mutations.

## 2. Results

### 2.1. Immunogenicity Assessment of Well-Known Driver Mutations

To identify neoantigens that might be commonly targeted for the treatment of various types of cancers, we assessed the immunogenicity of 10 well-known driver mutations, including KRAS-G12D, KRAS-G12V, KRAS-G12C, KRAS-G12R, KRAS-G13D, NRAS-Q61K, NRAS-Q61R, PIK3CA-E545K, PIK3CA-H1047R, and C-Kit-D816V. Ten long peptides (27-mer), each containing mutated amino acid residue located at the 12th to 14th positions from the N terminal, were synthesized so that both HLA class I- and class II-restricted T cell responses could be assessed (Table 1) and evaluated for their immunogenicity, using PBMCs from healthy volunteers possessing HLA-A*0201 or HLA-A*2402.

**Figure 1:**
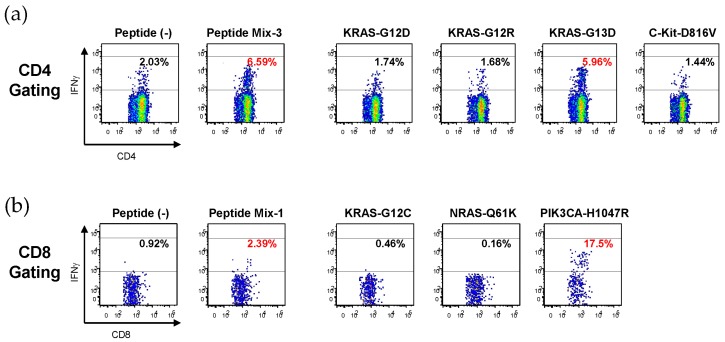
Representative flow cytometry profile of IFNγ production in CD4^+^ and CD8^+^ T cells. (**a**) Mix-3-stimulated CD4^+^ T cells (donor No. 21) were first examined for responses to the Mix-3, and then to each peptide constituent of the Mix-3. The percentages of IFNγ positive cells in CD4^+^ T cells are shown. (**b**) Mix-1-stimulated CD8^+^ T cells (donor No. 24) were first examined for responses to the Mix-1, and then to each peptide constituent of the Mix-1. The percentages of IFNγ positive cells in CD8^+^ T cells are shown.

Since there were limited numbers of available PBMCs, peptides that were pooled into three groups (Mix-1, Mix-2, and Mix-3; Table 2) were employed to stimulate the PBMCs. The peptide-stimulated cells were first evaluated for responses to the same peptide mixtures. If they were found to respond to a peptide mixture employed for stimulation, their precise antigen-specificity was confirmed by using each peptide constituent of the mixture. Figure 1 shows representative results. Mix-3-stimulated CD4^+^ T cells responded to Mix-3, and then to KRAS-G13D, one of the constituents of Mix-3, which suggests that KRAS-G13D might contain an HLA class II epitope (Figure 1a). Similarly, Mix-1-stimulated CD8^+^ T cells responded to Mix-1, and then to PIK3CA-H1047R, one of the constituents of Mix-1, which indicates that PIK3CA-H1047R might contain an HLA class I epitope (Figure 1b).

### 2.2. Summary of the Immunogenicity of 10 Well-Known Driver Mutations

The immunogenicity of 10 well-known driver mutations that were assessed using PBMCs from 25 normal donors is summarized in Table 2.

PBMCs stimulated with Mix-1 containing the KRAS-G12C-, NRAS-Q61K-, and PIK3CA-H1047R-derived peptides showed CD4^+^ and CD8^+^ T cell responses specific to Mix-1 in five and one donor(s), respectively. When Mix-1-stimulated cells from four donors were reanalyzed using each constituent peptide, only the PIK3CA-H1047R-derived peptide showed antigen-specific T cell responses. T cells from the remaining donor (No. 3) stopped growing during continued culturing and could not proceed to a detailed analysis. Overall, PIK3CA-H1047R immunogenicity was observed in four of the 24 donors (16.7%). This peptide induced CD4^+^ T cell responses, which might be HLA class II-restricted, in all four donors. Interestingly, it also induced CD8^+^ T cell responses, which might be HLA class I-restricted, in one donor (No. 24), which suggests that the PIK3CA-H1047R-derived peptide might contain both HLA class I- and class II-restricted epitopes.

PBMCs stimulated with Mix-2 containing the KRAS-G12V-, NRAS-Q61R-, and PIK3CA-E545K-derived peptides showed T cell responses specific to Mix-2 in six donors (CD4^+^ in three donors and CD8^+^ in three donors). Mix-2-stimulated cells derived from three of these six donors were successfully amplified and reanalyzed using each constituent peptide. As a result, only the NRAS-Q61R-derived peptide was found to induce antigen-specific CD4^+^ T cell responses, suggesting that it might contain an HLA class II-restricted epitope. Overall, the HLA class II-restricted immunogenicity of NRAS-Q61R was observed in three of the 24 donors (12.5%). By contrast, unfortunately, HLA class I-restricted immune responses specific to each peptide constituent of the Mix-2 were not confirmed.

PBMCs stimulated with Mix-3 containing the KRAS-G12D-, KRAS-G12R-, KRAS-G13D-, and C-Kit-D816V-derived peptides showed T cell responses specific to Mix-3 in seven donors (CD4^+^ in seven donors and CD8^+^ in one donor, respectively). When Mix-3-stimulated cells from all seven donors were reanalyzed using each constituent peptide, the KRAS-G12D-, KRAS-G12R-, KRAS-G13D-, and C-Kit-D816V-derived peptides showed antigen-specific CD4^+^ T cell responses in one (1/25, 4.0%), one (1/25, 4.0%), three (3/25, 12.0%), and four donors (4/25, 16.0%), respectively. The C-Kit-D816V-derived peptide also induced antigen-specific CD8^+^ T cell responses in one donor (1/25, 4.0%), suggesting that it might contain both HLA class I- and class II-restricted epitopes.

Taken together, six of the 10 driver mutations evaluated in this study, including KRAS-G12D, KRAS-G12R, KRAS-G13D, NRAS-Q61R, PIK3CA-H1047R, and C-Kit-D816V, induced antigen-specific CD4^+^ T cell responses, whereas two of the mutations (PIK3CA-H1047R and C-Kit-D816V) also induced antigen-specific CD8^+^ T cell responses. Notably, PIK3CA-H1047R, C-Kit-D816V, KRAS-G13D, and NRAS-Q61R seemed more immunogenic, since more than 10% of normal donors showed T cell responses to these mutations.

### 2.3. Determination of HLA Class II Restriction of T Cell Recognition for Peptides Derived from Driver Mutations

**Figure 2:**
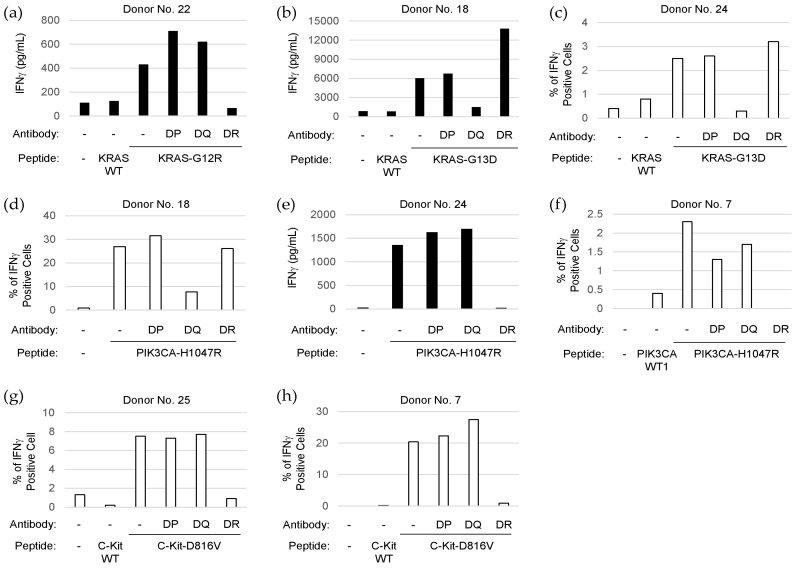
Determination of human leukocyte antigen (HLA) class II restriction of neoantigen-specific CD4^+^ T cells by blocking assay. Neoantigen-specific CD4^+^ T cells were stimulated without or with synthetic peptides (mutated or corresponding wild-type peptides) in the absence or presence of anti-HLA Class II antibody (anti-HLA-DP, -DQ, or -DR) to determine HLA class II restriction. Amounts of IFNγ secreted in the culture supernatant (closed bar) were determined by ELISA ((**a**) KRAS-G12R-specific cells from donor No. 22; (**b**) KRAS-G13D-specific cells from donor No. 18; and (**e**) PIK3CA-H1047R-specific cells from donor No. 24). The percentages of IFNγ positive cells in CD4^+^ T cells (open bar) were determined by flow cytometry ((**c**) KRAS-G13D-specific cells from donor No. 24; (**d**) PIK3CA-H1047R-specific cells from donor No. 18; (**f**) PIK3CA-H1047R-specific cells from donor No. 7; (**g**) C-Kit-D816V-specific cells from donor No. 25; and (**h**) C-Kit-D816V-specific cells derived from donor No. 7).

HLA class II restriction of TCR recognition for the peptides derived from driver mutations was assessed by stimulating antigen-specific T cells with the mutations in the presence of anti-HLA class II antibodies (Figure 2).

KRAS-G12R-specific T cells from a normal donor (No. 22) did not react with the corresponding wild-type peptide (KRAS-WT), confirming the immunogenicity of the mutated amino acid residue. The KRAS-G12R-specific responses were inhibited by anti-HLA-DR antibody, but not by anti-HLA-DP or anti-HLA-DQ antibody, indicating that the T cell response specific to KRAS-G12R might be HLA-DR-restricted (Figure 2a). By contrast, the KRAS-G13D-specific T cell responses were HLA-DQ-restricted in two donors (No. 18 and No. 24) (Figure 2b,c). Interestingly, the PIK3CA-H1047R-specific T cell responses were inhibited by anti-HLA-DQ antibody (donor No. 18) or anti-HLA-DR antibody (donor No. 7 and No. 24), suggesting that PIK3CA-H1047R could be recognized by T cells in both HLA-DQ- and HLA-DR-dependent manners (Figure 2d–f). The C-Kit-D816V-specific T cell responses were HLA-DR-restricted in two donors (No. 7 and No. 25) (Figure 2g,h).

**Figure 3:**
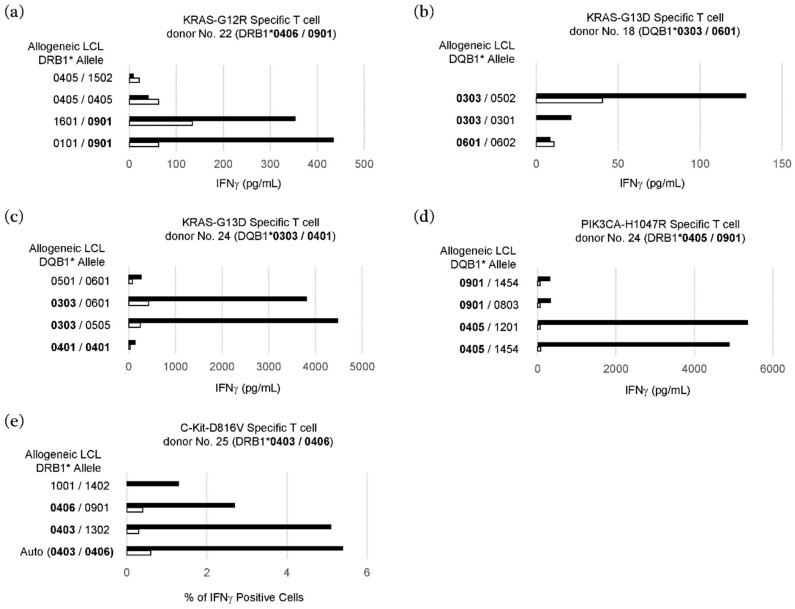
Determination of HLA class II restriction of neoantigen-specific CD4^+^ T cells by stimulation with the peptides presented by HLA class II-matched allogeneic lymphoblastoid cell lines (LCLs) as antigen presenting cells (APCs). HLA Class II-matched allogeneic LCLs were used as APCs to stimulate neoantigen-specific CD4^+^ T cells without (open bar) or with (closed bar) mutated synthetic peptides. HLA class II alleles of donors and matched LCLs are shown in bold. Amounts of IFNγ secreted in the culture supernatant by ELISA ((**a**) KRAS-G12R-specific cells from donor No. 22; (**b**) KRAS-G13D-specific cells derived donor No. 18; (**c**) KRAS-G13D-specific cells from donor No. 24; and (**d**) PIK3CA-H1047R-specific cells from donor No. 24) or percentages of IFNγ positive cells in CD4^+^ T cells by flow cytometry ((**e**) C-Kit-D816V-specific cells from donor No. 25) are shown.

To further confirm the HLA class II restriction of T cell recognition, specific T cells were stimulated with the peptides that were presented by HLA class II-matched allogeneic lymphoblastoid cell lines (LCLs) as antigen presenting cells (APCs) (Figure 3).

As shown in Figure 3a, the KRAS-G12R-specific T cells from donor No. 22 with HLA-DRB1*0406/0901 showed a substantial response to the KRAS-G12R-derived peptide only in the presence of allogeneic LCLs with HLA-DRB1*0901, which suggests that KRAS-G12R was recognized in an HLA-DRB1*0901-dependent manner. T cell responses specific to KRAS-G13D were detected in two donors (No. 18 and No. 24) who shared HLA-DQB1*0303. As expected, the KRAS-G13D-specific T cells responded to the KRAS-G13D-derived peptide only in the presence of allogeneic LCLs with HLA-DQB1*0303, which suggests the HLA-DQB1*0303-restricted recognition of KRAS-G13D (Figure 3b,c). On the basis of the co-culture of PIK3CA-H1047R-specific T cells from donor No. 24 with HLA-DRB1*0405/0901 and allogeneic LCLs with HLA-DRB1*0405, T cell recognition for PIK3CA-H1047R was HLA-DRB1*0405-restricted (Figure 3d). The C-Kit-D816V-specific T cells from donor No. 25 with HLA-DRB1*0403/0406 responded to the C-Kit-D816V-derived peptide in the presence of allogeneic LCLs with HLA-DRB1*0403 or HLA-DRB1*0406, suggesting that T cell recognition for C-Kit-D816V was restricted to both of these HLA-DRB1 alleles (Figure 3e).

### 2.4. Identification of Neoepitope Sequences from PIK3CA-H1047R and C-Kit-D816V

**Figure 4:**
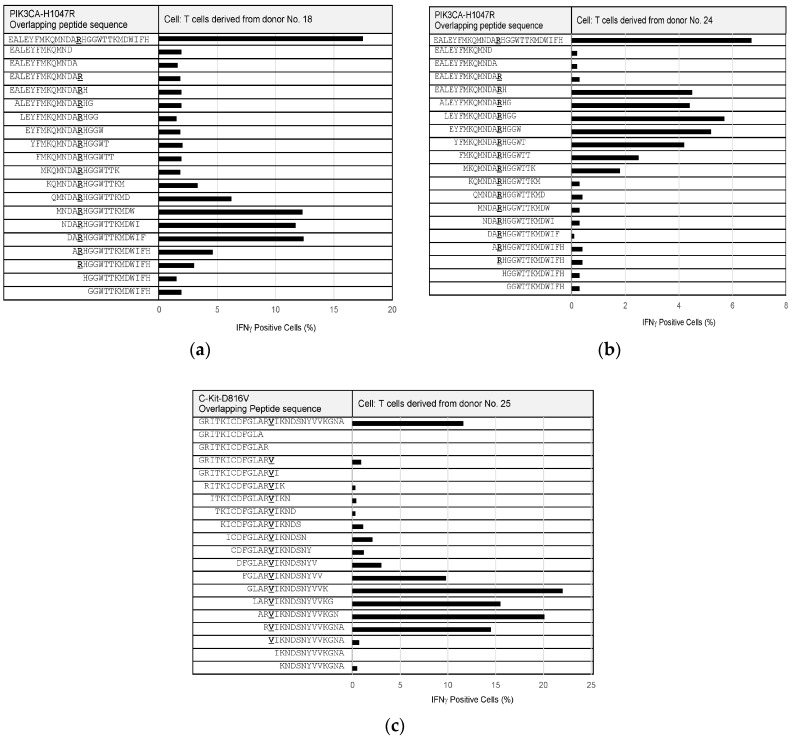
Epitope mapping of neoantigens. Overlapping peptides (12- to 15-mer) derived from PIK3CA-H1047R or C-Kit-D816V were tested for IFNγ production in CD4^+^ T cells specific to PIK3CA-H1047R or C-Kit-D816V, respectively. The percentages of IFNγ positive cells in neoantigen-specific CD4^+^ T cells were determined by flow cytometry after stimulation with each overlapping peptide, presented by autologous DCs as APCs. The sequences of overlapping peptides and percentages of IFNγ positive cells in CD4^+^ T cells are shown. (**a**) PIK3CA-H1047R-specific CD4^+^ T cells from donor No. 18; (**b**) PIK3CA-H1047R-specific CD4^+^ T cells from donor No. 24; and (**c**) C-Kit-D816V-specific CD4^+^ T cells from donor No. 25.

Since some of the PIK3CA-H1047R- and C-Kit-D816V-specific T cells could be maintained and expanded, we performed epitope mapping to precisely identify neoepitope sequences from these mutations (Figure 4). Overlapping peptides, of 12 to 15 amino acids in length, were synthesized to cover the full length of PIK3CA-H1047R- and C-Kit-D816V-derived 27-mer peptides and examined for their reactivity to the T cells specific to the corresponding mutations. The epitope that was recognized by PIK3CA-H1047R-specific T cells from donor No. 18 was composed of the following nine amino acids, “RHGGWTTKM”, (Figure 4a). In addition, another epitope from PIK3CA-H1047R was identified to consist of the nine amino acids “MKQMNDARH”, based on PIK3CA-H1047R-specific T cells from donor No. 24 (Figure 4b). The epitope that was recognized by the C-Kit-D816V-specific T cells from donor No. 25 was composed of the following 10 amino acids, “RVIKNDSNYV”, (Figure 4c).

A C-Kit-D816V-specific T cell clone (donor No. 25) was established by the limiting dilution method, and a single T cell from the clone was sorted into a 96-well plate. When the cDNAs of T cell receptors (TCRs) of eight different sorted single cells were sequenced, all of them showed completely the same sequences. PIK3CA-H1047R specific T cells were enriched by IFNγ secretion assay, and a single T cell from them was sorted into a 96-well plate. When the cDNAs of TCRs of sorted single cells were sequenced, 15 of 24 different single cells (63%) showed the same sequences. Representative TCR sequences of T cell clones specific to C-Kit-D816V and PIK3CA-H1047R are shown in Table 3.

## 3. Discussion

Considerable attention has recently been paid to T cell responses to neoantigens derived from tumor-specific genetic mutations, since tumors with many mutations have been demonstrated to be more likely to respond to immune checkpoint inhibitors than tumors with fewer mutations in cancer patients [7]. However, targeting neoantigens as personalized cancer immunotherapy is thought to be difficult and labor intensive, because most neoantigens are derived from “passenger mutations” that generally differ from patient to patient [5,6]. By contrast, neoantigens derived from “driver mutations” that are directly involved in cancer development and progression could represent appropriate off-the-shelf targets, because they are often shared by patients with various types of cancers. In addition, the loss of driver mutations as a result of immune editing and escape might be less likely to occur, since tumor cells must express driver genes to retain their malignant phenotypes. However, the immunogenicity of driver mutations has not yet been precisely investigated.

In the current study, we examined the T cell responses to 10 different driver mutations that are reported to commonly occur in various types of cancers. Among them, KRAS mutations (G12D, G12V, G12C, G12R, and G13D) are present at high frequencies in various cancer types including lung, colon, and pancreatic cancers [8]. Likewise, NRAS-Q61K and NRAS-Q61R mutations in skin and blood cancers [8]; PIK3CA-E545K and PIK3CA-H1047R mutations in breast, lung, and colon cancers [9]; and the C-Kit-D816V mutation in blood cancer [10] are reported to commonly and frequently occur. Since we could not predict T cell epitopes accurately and precisely [11], long synthetic peptides that may contain both HLA class I- and class II-restricted epitopes were employed for screening T cell responses. Although T cells from cancer patients with the corresponding mutations might be ideal for assessing their immunogenicity, it was difficult to obtain PBMCs from sufficient numbers of patients. Therefore, since previous reports showed that PBMCs from normal donors were useful for screening the immunogenicity of cancer-specific mutations [12], we used PBMCs from normal donors who possessed HLA-A*0201 or HLA-A*2402, which are the most popular types in Japan and Western countries. Since only limited numbers of PBMCs were available, three peptide mixtures were employed to stimulate the PBMCs. We selected peptides at random to make mixtures, because there have been no generalized rules to select them. Although it might be possible that selected peptides mutually affect each other for binding HLA molecules and/or stimulating antigen-specific T cells, we could not repeat the same experiments by using different combinations of peptides due to the limited availability of PBMCs.

The current study demonstrated that six of the 10 driver mutations, including KRAS-G12D, KRAS-G12R, KRAS-G13D, NRAS-Q61R, PIK3CA-H1047R, and C-Kit-D816V, induced antigen-specific CD4^+^ T cell responses. In addition, antigen-specific CD8^+^ T cell responses were also induced by PIK3CA-H1047R and C-Kit-D816V, indicating that they contain both HLA class I- and class II-restricted epitopes. Notably, immune-responses to PIK3CA-H1047R, C-Kit-D816V, KRAS-G13D, and NRAS-Q61R were observed in more than 10% of normal donors. Our results demonstrated that mutation-derived peptides could induce antigen-specific CD4^+^ T cells more frequently than CD8^+^ T cells. Consistent with our results, melanoma patients treated by personalized vaccination with long synthetic neoantigen peptides demonstrated that the proportion of neoantigens stimulating CD4^+^ T cell responses was higher than that for CD8^+^ T cell responses, despite the selection of mutations on the basis of predicted presentation by HLA class I [13]. Similarly, other reports also showed the induction of predominant CD4^+^ T cell responses after RNA vaccines targeting tumor-specific mutations in mouse models [14] and humans [15]. On the basis of these findings, the immunogenic mutations might be recognized by CD4^+^ T cells more preferentially than by CD8^+^ T cells. Such a predominance of CD4^+^ T cell responses might be explained by structural (promiscuous binding properties of peptides to MHC class II molecules) [16], cellular (difficulty processing long peptides or RNAs for cross-presentation to CD8^+^ T cells) [17], and other factors.

Since CD8^+^ cytotoxic T cells can directly kill tumor cells by recognizing antigen peptides in complexes with HLA class I molecules on the tumor cell surface, they have been thought to play a key role in anti-tumor immunity. Nevertheless, increasing attention has recently been paid to the roles of CD4^+^ T cells [18]. CD4^+^ T cells can induce potent anti-tumor immune responses in various ways, either indirectly by helping other immune cells, such as tumor-specific CTL and DCs, within the tumor microenvironment or directly by targeting tumor cells through cytolytic mechanisms [19]. For example, in murine tumor models, vaccination with MHC class II-restricted neoepitopes could induce strong anti-tumor activity [14]. In humans, the adoptive transfer of CD4^+^ T cells that recognize tumor-specific HLA class II-restricted neoantigens has been reported to show better tumor control in cancer patients [20,21]. Considering these results, the CD4^+^ T cell epitopes that were identified in this study could represent promising targets for patients with the corresponding mutations. For example, since we have already identified the TCR sequences from T cell clones specific to PIK3CA-H1047R and C-Kit-D816V, these sequences can be used to generate TCR-transduced T cells for adoptive transfer, as recently suggested [22]. In a future study, we will confirm that the TCR-transduced T cells can respond to cancer cells positive for the relevant mutations or APCs pulsed with lysates from them.

In the current study, the HLA class II restriction of four of the six immunogenic mutations was determined using anti-HLA blocking antibodies and a series of LCLs with different HLA types as APCs. Our results demonstrated that KRAS-G12R, KRAS-G13D, and PIK3CA-H1047R were restricted to HLA-DRB1*0901, DQB1*0303, and DRB1*0405, respectively, all of which are reported to be present in more than 10% of the Japanese population (DRB1*0901, 14.3%; DQB1*0303, 15.0%; DRB1*0405, 13.5%) [23,24]. This finding suggests that the identified mutations might be useful as off-the-shelf targets for personalized immunotherapy in Japanese patients. In addition, although we could not systematically and comprehensively screen the immunogenicity of the driver mutations due to the limited availability of PBMCs and the large diversity of HLA types, the current study showed that PIK3CA-H1047R contained at least two different CD4^+^ T cell epitopes restricted to different HLA alleles. These findings suggest that driver mutations are not always less immunogenic. Consistent with our results, other driver mutations, such as p53 [25,26], KRAS [26,27], BRAF [28], IDH1 [19], MYD88 [29], EZH2 [29], and histone 3 variant H3.3 [30], were reported to be immunogenic and induce antigen-specific CD4^+^ or CD8^+^ T cells. Conversely; however, recent reports have suggested that tumors are less likely to harbor driver mutations that bind well to MHC class I or class II, because mutations poorly bound to MHC class I or class II might be positively selected during tumorigenesis [31,32]. Further studies should more systematically and comprehensively assess and understand the immunogenicity of driver mutations. In particular, since this study determined the HLA-restriction and precise epitope sequences in PIK3CA-H1047R or C-Kit-D816V, we will confirm their immunogenicity in T cells from HLA-matched cancer patients with the corresponding mutations in a future study.

## 4. Materials and Methods

### 4.1. Cells and Peptides

Human PBMCs from normal donors (*n* = 15; donor No. 1 to 15) positive for HLA-A*24:02 or A*02:01 were purchased from Precision Medicine Group, Inc. (Austin, TX, USA). In addition, PBMCs positive for HLA-A*24:02 or A*02:01 were also obtained from the peripheral blood of 10 healthy volunteers (donor No. 16 to 25) by density gradient centrifugation (Lymphoprep; Axis-Shield, Dundee, Scotland) at the Kanagawa Cancer Center Research Institute; these PBMCs were cryopreserved with Cellbanker1 (Nippon Zenyaku Kogyo Co.,Ltd., Tokyo, Japan) at −80 °C until use. The HLA types were determined via the next generation sequencing method at the HLA Laboratory (Kyoto, Japan). A series of LCLs with different HLA types was prepared by infecting non-adherent cells from PMBCs with the culture supernatant of Epstein-Barr (EB) virus-producing cells (B95-8 cells; JCRB Cell Bank, JCRB 9123); these LCLs were used as APCs for T cell stimulation. All healthy volunteers gave their informed consent for inclusion before they participated in the study. The study was conducted in accordance with the Declaration of Helsinki, and the protocol was approved by the Ethics Committee of Kanagawa Cancer Center (Project identification code 27-7).

Synthetic peptides (27-mer) containing the amino acid sequences derived from 10 known driver mutations, including KRAS-G12D, KRAS-G12V, KRAS-G12C, KRAS-G12R, KRAS-G13D, NRAS-Q61K, NRAS-Q61R, PIK3CA-E545K, PIK3CA-H1047R, and C-Kit-D816V, and their corresponding wild-type sequences were provided at purities greater than 80% by Merck KGaA (Darmstadt, Germany). The mutated amino acid residues were located at the 12th to 14th positions from the N terminal. Overlapping synthetic peptides (12- to 15-mer) derived from PIK3CA-H1047R or C-Kit-D816V were also synthesized at purities greater than 80% (Merck KGaA). The lyophilized powder of the peptides was dissolved in dimethyl sulfoxide (Merck KGaA) at a concentration of 10 mg/mL and stored at −20 °C until use.

### 4.2. PBMC Stimulation for the Induction of Antigen-Specific T Cells

PBMCs (2 × 10^6^ cells) were cultured in AIM-V medium (Thermo Fisher Scientific K. K., Tokyo, Japan) supplemented with 5% heat-inactivated human serum (MP Biomedicals, Santa Ana, CA, USA) for 7 days in the presence of peptide mixture (2 µg/mL each) at 37 °C. Simultaneously, the adherent fraction of the PBMCs from the same donors was cultured in AIM-V with 50 ng/mL granulocyte macrophage colony-stimulating factor (GM-CSF; PeproTech, Inc., Rocky Hill, NJ, USA) and 50 ng/mL IL-4 (PeproTech, Inc.) for 7 days to generate immature dendritic cells (DCs). After culturing for 7 days, the peptide-stimulated PBMCs were collected and co-cultured with mitomycin C (Kyowa Hakko Kirin Co., Ltd., Tokyo, Japan)-treated autologous DCs (1 × 10^5^ cells) in the presence of the same concentration of peptides and 0.1 KE/mL OK-432 (Picibanil for injection, Chugai Pharmaceutical Co., Ltd., Tokyo, Japan), followed by the addition of IL-2 (10 IU/mL; PeproTech Inc.) on the 9th day. On the 14th day, the peptide-stimulated cells were re-stimulated with MMC-treated autologous DCs (1 × 10^5^) pulsed with the same concentration of peptides. On the 21st day, the cells were examined for antigen-specific IFNγ production by intracellular IFNγ staining or an IFNγ ELISA.

### 4.3. Intracellular IFNγ Staining

Peptide-stimulated cells (5.0 × 10^4^ cells) were co-cultured with autologous DCs (5 × 10^3^ cells) in a 96-well U-bottom plate (Corning Incorporated, Corning, NY, USA) in the absence or presence of a single peptide (5 µg/mL) or peptide mixture (2 µg/mL each). For the intracellular cytokine staining, 10 µg/mL of Brefeldin A (Merck KGaA) was added 2 h after the culture was initiated. After culturing for an additional 20–24 h, the cells were stained with APC-labeled anti-CD3 (Clone UCHT1; Biolegend, San Diego, CA, USA), FITC-labeled anti-CD4 (Clone RPA-T4; Becton, Dickinson and Company, Franklin Lakes, NJ, USA), and APC-Cy7-labeled anti-CD8 (Clone RPA-T8; TONBO Biosciences, San Diego, CA, USA) antibodies for 15 min at 4 °C. After washing, they were fixed and permeabilized with BD Cytofix/Cytoperm (Becton, Dickinson and Company) for 20 min at 4 °C, and then stained with PE-Cy7-labeled anti-IFNγ antibody (Clone B27, Becton, Dickinson and Company) for 40 min at 4 °C. After washing, the samples were run on a FACSCanto II (Becton, Dickinson and Company), and the data were analyzed to determine the percentages of IFNγ-positive cells in CD4- or CD8-positive cells by using the FACSDiva^TM^ software (Becton, Dickinson and Company). If the percentages were 1.3% or higher and were also 1% or greater than the controls without peptide treatment, they were defined as positive. In some experiments, peptide stimulation was performed in the presence of anti-HLA class II antibodies (200 µg/mL anti-HLA-DP (Clone BRAFB6; Santa Cruz Biotechnology Inc., Santa Cruz, CA, USA), 1 mg/mL anti-HLA-DQ (Clone SPV-L3; Abcam PLC, Cambridge, UK), or 1 mg/mL anti-HLA-DR (Clone G46-6; Becton, Dickinson and Company)) to block HLA class II-restricted T cell recognition. To further determine the HLA restriction of peptide-stimulated T cells, a series of LCLs with different HLA types was used at the LCL: T cell ratio of 2:1 (LCL, 2 to 10 × 10^4^ cells/well vs. T cells, 1 to 5 × 10^4^ cells/well).

### 4.4. IFNγ ELISA

Peptide-stimulated cells (5.0 × 10^4^ cells) were co-cultured with autologous DCs (5 × 10^3^ cells) in a 96-well U-bottom plate (Corning Incorporated) in the absence or presence of a single peptide (5 µg/mL) or peptide mixture (2 µg/mL each). After 24 h, the culture supernatants were collected and analyzed for IFNγ secretion by an ELISA, according to the manufacturer’s instructions (Becton, Dickinson and Company). Briefly, ELISA plates were coated with anti-IFNγ capture antibody overnight at 4 °C and blocked with Assay Diluent at room temperature for 1 h. After washing, the samples (1- to 10-fold dilution) were added and incubated at room temperature for 90 min. After washing, a mixture of biotinylated anti-IFNγ detection antibody and streptavidin-horseradish peroxidase conjugate was added, and the solution was incubated at room temperature for 45 min. After washing and adding TMB substrate solution, the reaction was stopped with Stop Solution and the absorbance was measured at O.D. 450 nm with an ARVO^TM^ X4 plate reader (PerkinElmer Inc., Waltham, MA, USA). In some experiments, peptide stimulation was performed in the presence of anti-HLA class II (anti-HLA-DP, anti-HLA-DQ, or anti-HLA-DR) antibodies, or a series of LCLs with different HLA types were used as APCs to determine the HLA-restriction, as mentioned above.

### 4.5. Determination of TCR Sequences of Antigen-Specific T Cell Clones

C-Kit-D816V-stimulated T cells were plated by limiting dilution (0.5–2.0 cells/well) and expanded in a 96-well U-bottom plate (Becton, Dickinson and Company) in AIM-V medium supplemented with 5% heat-inactivated human serum, 40 ng/mL anti-CD3 antibody (Clone UCHT 1; Becton, Dickinson and Company), and 120 IU/mL IL-2 (PeproTech, Inc.). The expanded clones were examined for IFNγ production after peptide stimulation, and a C-Kit-D816V-specific T cell clone was obtained. A single T cell from the obtained clone was sorted into a 96-well plate (4titude, Wotton, UK) by the FACSAria II cell sorter (Becton, Dickinson and Company). PIK3CA-H1047R specific T cells were enriched by IFNγ secretion assay (Miltenyi Biotec, Auburn, CA, USA), followed by sorting of a single T cell into a 96-well plate (4titude) by the FACSAria II cell sorter (Becton, Dickinson and Company).

The sequences of TCR genes of the sorted T cell clones were determined, as reported previously [33]. The cDNA of TCRα and TCRβ chain was amplified from single cells and sequenced at Eurofin Genomics K.K. (Tokyo, Japan).

## 5. Conclusions

In summary, the current study showed that frequent driver mutations, including KRAS-G12D, KRAS-G12R, KRAS-G13D, NRAS-Q61R, PIK3CA-H1047R, and C-Kit-D816V, induced antigen-specific T cell responses. Since these driver mutations are shared by patients with various types of cancers and are not easily lost via immune escape, they have the potential to be promising off-the-shelf targets of tumor immunotherapy against patients with the corresponding mutations. More detailed analyses and information should be provided to accelerate the clinical application of personalized immunotherapy targeting such frequent driver mutations.

## Figures and Tables

**Table 1:** List of the synthetic peptides derived from driver mutations.

Driver Mutation		Peptide Analyzed in This Study	
Gene	Mutation	Name	Peptide Sequence ^1^
KRAS	(Wild-Type)	KRAS-WT	MTEYKLVVVGAGGVGKSALTIQLIQNH
	G12D	KRAS-G12D	MTEYKLVVVGA**D**GVGKSALTIQLIQNH
	G12V	KRAS-G12V	MTEYKLVVVGA**V**GVGKSALTIQLIQNH
	G12C	KRAS-G12C	MTEYKLVVVGA**C**GVGKSALTIQLIQNH
	G12R	KRAS-G12R	MTEYKLVVVGA**R**GVGKSALTIQLIQNH
	G13D	KRAS-G13D	MTEYKLVVVGAG**D**VGKSALTIQLIQNH
NRAS	(Wild-Type)	NRAS-WT	GETCLLDILDTAGQEEYSAMRDQYMRT
	Q61K	NRAS-Q61K	GETCLLDILDTAG**K**EEYSAMRDQYMRT
	Q61R	NRAS-Q61R	GETCLLDILDTAG**R**EEYSAMRDQYMRT
PIK3CA	(Wild-Type)	PIK3CA-WT1	EALEYFMKQMNDAHHGGWTTKMDWIFH
	H1047R	PIK3CA-H1047R	EALEYFMKQMNDA**R**HGGWTTKMDWIFH
	(Wild-Type)	PIK3CA-WT2	KAISTRDPLSEITEQEKDFLWSHRHYC
	E545K	PIK3CA-E545K	KAISTRDPLSEIT**K**QEKDFLWSHRHYC
C-Kit	(Wild-Type)	C-Kit-WT	GRITKICDFGLARDIKNDSNYVVKGNA
	D816V	C-Kit-D816V	GRITKICDFGLAR**V**IKNDSNYVVKGNA

^1^ Mutated position is bold and underlined.

**Table 2:** Summary of immunogenicity of the synthetic peptides derived from driver mutations.

Donor No.	Mix-1 ^1^				Mix-2 ^1^				Mix-3 ^1^				
		KRAS- G12C	NRAS- Q61K	PIK3CA- H1047R		KRAS- G12V	NRAS- Q61R	PIK3CA- E545K		KRAS- G12D	KRAS- G12R	KRAS- G13D	C-Kit D816V
1	-				CD4				-				
2	-				-				-				
3	CD4 ^2^				-				-				
4	-				-				-				
5	-				-				-				
6	-				-				-				
7	CD4	-	-	CD4	CD8 ^3^	-	CD4	-	CD4	-	-	-	CD4
8	-				-				-				
9	-				-				-				
10	-				-				-				
11	-				-				-				
12	-				-				-				
13	-				CD8				-				
14	-				-				-				
15	-				CD8				-				
16	-				-				-				
17	-				-				-				
18	CD4	-	-	CD4	-				CD4	-	-	CD4	-
19	-				-				-				
20	-				-				CD4	-	-	-	CD4
21	-				-				CD4	-	-	CD4	-
22	-				CD4	-	CD4	-	CD4	-	CD4	-	CD4
23	CD4	-	-	CD4	-				-				
24	CD4/8	-	-	CD4/8	CD4	-	CD4	-	CD4/8	CD4	-	CD4	CD8
25									CD4	-	-	-	CD4
Frequency				16.7% (4/24)			12.5% (3/24)			4.0% (1/25)	4.0% (1/25)	12.0% (3/25)	20.0% (5/25)

^1^ Mix-1—KRAS-G12C, NRAS-Q61R, and PIK3CA-H1047R; Mix-2—KRAS-G12V, NRAS-Q61R, and PIK3CA-E545K; Mix-3—KRAS-G12D, KRAS-G12R, KRAS-G13D, and C-Kit-D816V. ^2^ Antigen-specific CD4^+^ T cells detected by intracellular interferon-gamma (IFNγ) staining. ^3^ Antigen-specific CD8^+^ T cells detected by intracellular IFNγ staining. (-)—not detected; (blank)—not tested.

**Table 3:** T cell receptor (TCR) sequences of T cell clones specific to PIK3CA-H1047R and C-Kit-D816V.

Antigen	TCR	Variable (V)	Joining (J)	Diversity (D)	CDR3 ^1^
C-Kit- D816V	TRA	TRAV1-1	TRAJ39	-	CAVRDNAGNMLTF
	TRB	TRBV19	TRBJ1-2	TRBD2	CASSIPNLGYGYTF
PIK3CA- H1047R	TRA	TRAV23/DV6	TRAJ43	-	CAASGSYNNNDMRF
	TRB	TRBV6-5	TRBJ2-2	TRBD1	CASSYASPGTGYSGELFF

^1^ CDR3, complementarity–determining region 3.
